# ANEMIA, MELENA, AND AN INCONCLUSIVE BIOPSY: A CHALLENGING CECAL MASS CASE

**DOI:** 10.51894/001c.123075

**Published:** 2024-08-30

**Authors:** Gillian Tyner

**Affiliations:** 1 Internal Medicine Detroit Medical ICenter Sinai Grace Hospital

## 73

### INTRODUCTION

Overt upper and lower gastrointestinal (GI) tract bleeding can encompass a broad spectrum of pathophysiologic processes including, but not limited to, erosive, hematologic, inflammatory, oncologic, traumatic, and vascular etiologies. Overt GI bleeding is associated with various clinical features, including hematochezia, melena, anemia, tachycardia, presyncope, and more. Upper GI bleeding usually presents with hematemesis and/or melena. Hematochezia usually indicates bleeding from the lower GI tract. The initial diagnostic workup depends on the patient’s condition and may indicate the source of bleeding in either the upper or lower GI tract. Hemodynamically stable patients suspected of having an upper GI bleed undergo an esophagogastroduodenoscopy (EGD), while hemodynamically stable patients suspected of having a lower GI bleed undergo a colonoscopy.

### CASE DESCRIPTION

A 65-year-old man with a medical history of gastritis and antral ulcers presented to the hospital with a chief complaint of melena associated with fatigue and palpitations for an unknown duration of time. On presentation, physical examination was significant for hypotension, tachycardia, pale conjunctiva, and epigastric pain. The patient’s hemoglobin was initially 4.5 gm/dL. CT abdomen/pelvis with and without contrast demonstrated bowel wall thickening involving the mid-distal descending colon. Gastroenterology was consulted, an EGD was performed, was significant for non-bleeding esophageal varices, and the source of melena was not identified. The patient never had a screening colonoscopy, the patient was prepared for colonoscopy, and the colonoscopy was significant for a nodular, ulcerative, friable, and non-obstructing cecal mass. The cecal mass was biopsied, results were negative for malignancy, and results were predominantly composed of granulation tissue. Colorectal surgery was consulted, metastatic workup was negative, the patient underwent a robotic-assisted right hemicolectomy with extracorporeal anastomosis, and the final pathology result was a 4 cm sessile serrated adenoma with extensive ulceration.

### CONCLUSION

The patient’s overt GI tract bleeding source was identified following hemicolectomy. This case reinforces that melena can be caused by bleeding in the upper and lower GI tract. This case highlights the importance of screening colonoscopies to detect colon polyps and prevent the development of high-risk features, which, in turn, can limit aggressive treatment strategies such as hemicolectomies, as seen in this case.

